# Corneal Epithelial Microcysts due to High-Dose Cytarabine Administration in a Pediatric Acute Myeloid Leukemia Patient

**DOI:** 10.5505/tjh.2012.21704

**Published:** 2012-03-05

**Authors:** Tuba Hilkay Karapınar, Salih Gözmen, Özlem Tüfekçi, Şebnem Yılmaz, Zeynep Özbek, Melih Parlak, Gülersu İrken, Hale Ören

**Affiliations:** 1 Dokuz Eylul University, School of Medicine, Department of Pediatric Hematology, Balcova, Izmir, Turkey; 2 Dokuz Eylul University, School of Medicine, Department of Ophthalmology, Balcova, Izmir, Turkey

## TO THE EDITOR

Over the past 2 decades the advent of new therapeutic strategies has led to remarkable progress in the treatment of acute myeloid leukemia (AML ) [1]; however, some serious side effects of treatment may be clinically apparent [2]. An 8-year-old boy was diagnosed as AML and started on AML -BFM 2004 therapy in August 2009. There were no important side effects observed until the initiation of hAM block treatment (1 g m–2 of cytarabine every 12 h on d 1-3 and 10 mg·m–2·d–1 of mitoxantrone on d 3). We have written informed consent.

**Figure 1:**
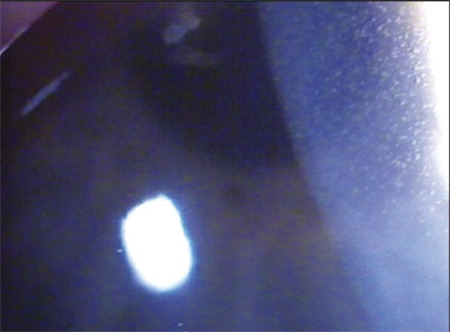
Photograph depicting multiple corneal epithelial microcysts.

**Figure 2:**
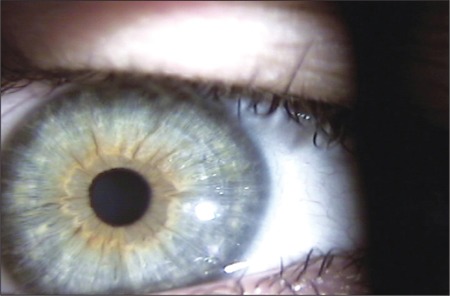
Photograph shows clear cornea 1 month after thetreatment.

On d 6 of hAM block treatment the patient complained of a burning sensation in his eyes and photophobia. Ophthalmologic consultation showed bilateral multiple diffuse corneal epithelial microcysts and mild superficial punctate epitheliopathy (Figure 1,2). The remainder of the ophthalmologic examination was normal. Conservative management with topical artificial teardrops and followup was planned. The patient’s ophthalmologic complaints reduced in severity within 5 d and the corneal findings decreased and disappeared within 3 weeks.

The patient’s next block treatment was HAE block and consisted of a higher dose of cytarabine (3 g m–2 every 12 h on d 1-3) than did the haM block treatment (1 g m–2 cytarabine), and also included 125 mg·m–2·d–1 of etoposide on d 2-5. High-dose cytarabine (1 g 100 mL^–1^ concentration in 5% dextrose) was given for 3 h this time. Topical artificial tears were continued every 2 h together with artificial tear gel b.i.d. Photophophia and the burning sensation in the patient’s eyes recurred, but resolved 3 d after drug cessation.

Systemic medications may reach the cornea via the tear film, aqueous humor, or limbal vasculature [3]. Corneal changes may result in reduced visual acuity, photophobia, and ocular irritation—symptoms that typically resolve following drug cessation. Ocular side effects of systemic medications are often dose-related and transient. High doses of systemic drugs may lead to progressive lenticular changes as well as irreversible retinal toxicity [3].

Cytarabine is a cell cycle-specific (S phase) antimetabolite that inhibits DNA synthesis. Cytarabine exerts its greatest effect on rapidly dividing cells, and may be toxic to the corneal epithelium, despite its use as a topical agent [2]. Systemic use at high doses may also produce corneal and conjunctival epithelial toxicity, with conjunctival hyperemia, punctate keratopathy, and corneal epithelial microcysts [4].

Histopathologic examination shows profound degeneration of the rapidly dividing basal epithelial cells, which leads to formation of epithelial microcysts. Cytarabine may be administered intravenously or intrathecally, with ocular symptoms typically developing within 1 week of initiation. Cytarabine penetrates the blood-brain barrier following intravenous injection and may affect the cornea via both the aqueous humor and tears. Visual symptoms include tearing, photophobia, foreign body sensation, pain, and reduced visual acuity [4]. It is suggested that ocular toxicity results from the inhibition of corneal epithelial DNA synthesis, and is related to both drug dosage and duration of use [5]. We presented a case of corneal epithelial microcysts associated with high-dose cytarabine treatment. Clinicians should consider corneal epithelial microcysts in patients that experience tearing, photophobia, pain, and reduced visual acuity following cytarabine treatment.

## CONFLICT OF INTEREST STATEMENT

The authors of this paper have no conflicts of interest, including specific financial interests, relationships, and/ or affiliations relevant to the subject matter or materials included.
